# Associations of sitting time and occupation with metabolic syndrome in South Korean adults: a cross-sectional study

**DOI:** 10.1186/s12889-016-3617-5

**Published:** 2016-09-07

**Authors:** Jin Young Nam, Juyoung Kim, Kyung Hee Cho, Young Choi, Jaewoo Choi, Jaeyong Shin, Eun-Cheol Park

**Affiliations:** 1 Department of Public Health, Graduate School, Yonsei University, Seoul, Republic of Korea; 2 Institute of Health Services Research, Yonsei University, Seoul, Republic of Korea; 3 Department of Preventive Medicine, Yonsei University College of Medicine, Seoul, Republic of Korea

**Keywords:** Sitting-time, Sedentary behavior, Occupation, Metabolic syndrome

## Abstract

**Background:**

Previous evidence suggests that there is a correlation between prolonged sitting time and cardio-metabolic disease, such as metabolic syndrome (MS). Cardiovascular disease is the second-leading cause of mortality in South Korea, a country with the longest working hours among all member states of the Organization for Economic Co-operation and Development. However, no previous study has investigated the relationships of overall sitting-time and occupation with MS in South Korea. Accordingly, the present study examined these relationships in a South Korean population.

**Methods:**

Data from the sixth Korean National Health and Nutrition Examination Survey (KNHANES), a nationally representative survey with a cross-sectional design, were used in the present study. MS diagnoses were evaluated using the International Diabetes Foundation (IDF) criteria. Participants self-reported their overall sitting times, and occupations were classified using the Korean version of the Standard Classification of Occupations (KSCO). A multiple logistic regression analysis was conducted to evaluate the associations of sitting time and occupation with MS.

**Results:**

The risk of MS was 1.21-fold higher among participants who sat for >7 h/day than among those who sat for ≤7 h/day (odds ratio [OR]: 1.21, 95 % confidence interval [CI]: 1.00–1.46). Regarding occupation, office workers had a two-fold higher risk of MS than did agriculture, forestry, and fishery (AFF) workers (OR: 2.01, 95 % CI: 1.26–3.22). In a combined analysis of sitting time and occupation, male participants who sat for >7 h/day and reported an occupation that involves office work (OW) or machine fitting (MF) were significantly more likely to have MS when compared to those who sat for ≤7 h/day and were employed as AFF workers (>7 h/day × OW, OR: 2.41, 95 % CI: 1.05–5.51; >7 h/day × MF, OR: 2.92, 95 % CI: 1.43–5.93).

**Conclusions:**

Excessive sitting time and a sedentary occupation correlated positively with MS in South Korean adults. Accordingly, a reduction in the overall sitting time or inclusion of energy-expending activities in the workplace might improve the rate of MS.

## Background

Metabolic syndrome (MS), one of the most widely used markers of cardio-metabolic risk, affects cardiovascular morbidity and mortality, as well as all-cause mortality [1] and is gradually increasing in prevalence worldwide. According to data from the National Health and Nutrition Examination Survey (NHANES) of 1999–2006, the age-adjusted prevalence of MS increased from 29.2 to 34.2 % in the USA during the survey period [2], and similar trends have been observed in Asian countries [3]. Notably, South Korea reported an increase in the age-adjusted prevalence of MS from 29.2 to 31.3 % during the period of 1998–2007 [4], suggesting a possible increase in the risk of cardiovascular disease. The sharp increase in the rate of cardiovascular disease–associated mortality from 35.6 to 52.4 out of 100,000 people in South Korea between 2003 and 2014 [5] underscores the need to reduce the prevalence of conditions that lead to cardiovascular disease, such as MS.

Sedentary behavior, defined as participation in non-physical activities that do not require significant energy expenditure above that required for rest or for sedentary activities such as sitting, lying down, watching television, reading books, and using computers [6, 7], has recently been identified as a significant public health issue. In addition, several epidemiologic studies have suggested a relationship between sedentary behavior, distinguished from a mere lack of physical activity, and adverse health outcomes such as obesity, diabetes, insulin resistance, MS, cardiovascular disease, cancer, and death [6–8]. Currently, working adults spend approximately one third to a half of their workday in a seated position [9–11] and spend hours of leisure time on activities such as watching TV, using computers, participating in screen-based recreation, and driving [12, 13]. Previous studies conducted in 20 countries worldwide found that all participants except those in Africa sat for 5.8 h/day [14], adults in the USA were sedentary for 7.3–7.9 h/day [15], and adults in Australia were sedentary for an average of 57 % of their waking time [16]. In other words, many people spend a great deal of their waking time in the workplace [17, 18]. Accordingly, interventions have indicated that workplace health promotion programs that target sedentary behavior and promote physical activity (PA) could encourage employees to participate in the latter during their breaks from work [19–21]. Unfortunately, the working hours reported by Korean adults are among the longest recorded for citizens of member countries of the Organization for Economic Co-operation and Development (OECD) [22]; thus, people likely spend their most of their waking hours at the workplace and are therefore easily exposed to adverse health outcomes related to prolonged sitting times, such as MS.

Therefore, this cross-sectional study evaluated the relationships of sitting time and occupation with MS in a nationally representative sample of South Korean adults. In this study, the reference of sitting time was the median value of total study population. This study aimed to examine the associations of these factors and estimate differences in the prevalence of MS according to the combination of occupation and overall sitting time.

## Methods

### Participant and database information

This study used data collected during the second year (2014) of the sixth Korean National Health and Nutrition Examination Survey (KNHANES), which was conducted by the Korea Centers for Disease, Control, and Prevention of South Korea (KCDC). The KNHANES is a cross-sectional, nationally representative survey that has been conducted regularly since 1998 to examine the general health and nutritional status of Korean citizens. The initial sample included 9701 individuals aged >1 year who were invited to participate in the second year of the sixth KNHANES. A total of 7550 individuals participated in the survey (response rate: 77.8 %). The dataset supporting the conclusions of this article is available in the KNHANES repository (https://knhanes.cdc.go.kr/knhanes/index.do).

The present study included adults (age: ≥19 years) who responded to the survey in 2014. Individuals who were younger than 18 years (*n* = 1408) and for whom no information about MS, sitting times, and covariates were available (*n* = 1673) were excluded. Ultimately, a total of 4303 individuals (1739 men, 2564 women) were deemed eligible.

### Measures

#### Definition of metabolic syndrome

The presence of MS was measured using the International Diabetes Foundation (IDF) criteria [23]. According to the criteria, a patient was required to have central obesity (defined for Koreans as a waist circumference of ≥90 cm in men and ≥85 cm in women [24]) plus any two of the following four factors: (1) an increased triglyceride level of ≥150 mg/dL or specific treatment; (2) a decreased high-density lipoprotein cholesterol level of <40 mg/dL in men and <50 mg/dL in women or specific treatment; (3) raised blood pressure, indicated by a systolic blood pressure of ≥130 mmHg, a diastolic blood pressure of ≥85 mmHg, or treatment of previously diagnosed hypertension; and (4) an increased fasting plasma glucose level of ≥100 mg/dL or previously diagnosed type 2 diabetes.

#### Overall sitting-time per day: International Physical Activity Questionnaire

The overall daily sitting time was estimated using the long-version of the International Physical Activity Questionnaire [25, 26], particularly the following question: *How many hours do you typically spend sitting or lying down while engaged in activities such as working at a desk or computer, visiting friends, driving, reading, writing, watching television, playing games, using the Internet, or listening to music on a usual day?* Responses were divided into two categories according to the median value: *≤7 h/d* and *>7 h/d.*

#### Occupation

Specific occupations were classified into nine major categories according to the Korean version of the Standard Classification of Occupations (KSCO), based on the International Standard Classification of Occupations by the International Labor Organization. For this study, several of the nine categories were combined to yield the following five categories: *office work (OW), sales and services (SS), agriculture, forestry and fishery (AFF), machine fitting and simple labor (MF), and unemployed (including housewives and students).*

#### Covariates

Sex, age, household income, and educational levels were considered as socioeconomic factors. Health-related factors included measures of physical activity, energy intake, depressive symptoms, current smoking status, frequency of alcohol use, and number of chronic diseases. PA was measured according to the performance of aerobic activity as recommended by the WHO guidelines [27]. Those who participated in at least 150 min of moderate-intensity PA per week, at least 75 min of vigorous-intensity aerobic activity per week, or combinations of moderate- and vigorous-intensity activities (e.g., 1 min of intense activity and 2 min of moderately intense activity) for at least 75 min per week were classified as “meet recommended guidelines”; all others were classified as “under meet recommended guidelines.” The energy intakes were divided into four categories according to quartile values: 1Q was the lowest energy intake and 4Q was the highest. The current smoking status was based on the definition of current cigarette smoking status used in the National Health Interview Survey (NHIS) [28]. It was dichotomized as current smokers plus those who had smoked more than 100 cigarettes during their lifetimes versus never smokers and those who had previously smoked less than 100 cigarettes during their lifetimes. The frequency of alcohol use was based on the definition of current alcohol drinking status used in the NHIS [28]. It was measured via queries on the participants’ average alcohol consumption frequency (more than once per month or not/never drinking) during the previous year. Three categories were used to classify participants according to the presence of zero, one, or two or more chronic diseases, which included stroke, myocardial infarction, angina, arthritis, rheumatoid arthritis, chronic renal failure, asthma, thyroid disease, and hepatitis B.

#### Statistical analysis

General characteristics were evaluated using the chi-square test, and multiple logistic regression models were used to estimate potential correlations of sitting time and occupation with MS. In addition, the interaction between sitting time and occupation was observed to affect MS, and a combined multiple logistic regression analysis was performed accordingly. All statistical analyses were conducted using SAS 9.4 (SAS Institute, Inc., Cary, NC, USA).

## Results

Table 1 presents the participants’ general characteristics. The 4303 participants included 583 (13.6 %) with MS—307 men (17.7 %) and 276 women (10.8 %)—and 3720 (86.4 %) without MS. Furthermore, 45.8 % of participants reported a sitting time >7 h/day, and a higher proportion of MS was observed among those who sat for >7 h/day (13.9 %) versus those who sat for ≤7 h/day (13.3 %).

**Table 1:** General characteristics of the study population

	Metabolic syndrome						
	Total		Yes		No		
	*N*	(%)	*N*	(%)	*N*	(%)	*p*-value
Sitting time (hours)							
≤ 7	2332	(54.19)	310	(13.29)	2022	(86.71)	0.5944
> 7	1971	(45.81)	273	(13.85)	1698	(86.15)	
Sex							
Male	1739	(40.41)	307	(17.65)	1432	(82.35)	<0.0001
Female	2564	(59.59)	276	(10.76)	2288	(89.24)	
Age (year)							
19–29	474	(11.02)	11	(2.32)	463	(97.68)	<0.0001
30–39	758	(17.62)	59	(7.78)	699	(92.22)	
40–49	741	(17.22)	77	(10.39)	664	(89.61)	
50–59	834	(19.38)	119	(14.27)	715	(85.73)	
60–69	787	(18.29)	170	(21.60)	617	(78.40)	
70+	709	(16.48)	147	(20.73)	562	(79.27)	
Household income level							
Low	790	(18.36)	154	(19.49)	636	(80.51)	<0.0001
Lower-middle	1076	(25.01)	149	(13.85)	927	(86.15)	
Upper-middle	1255	(29.17)	156	(12.43)	1099	(87.57)	
High	1182	(27.47)	124	(10.49)	1058	(89.51)	
Educational level							
Elementary school	1007	(23.40)	230	(22.84)	777	(77.16)	<0.0001
Middle school	487	(11.32)	79	(16.22)	408	(83.78)	
High school	1411	(32.79)	155	(10.99)	1256	(89.01)	
College or higher	1398	(32.49)	119	(8.51)	1279	(91.49)	
Occupation							
Office work	939	(21.82)	101	(10.76)	838	(89.24)	0.0024
Sales and Service	516	(11.99)	56	(10.85)	460	(89.15)	
Agriculture, forestry, and fishery	242	(5.62)	36	(14.88)	206	(85.12)	
Machine fitting and simple labor	775	(18.01)	128	(16.52)	647	(83.48)	
Unemployed, housewife, or student	1831	(42.55)	262	(14.31)	1569	(85.69)	
Physical activity							
Under meet recommended guidelines	2032	(47.22)	302	(14.86)	1730	(85.14)	0.0172
Meet recommended guidelines	2271	(52.78)	281	(12.37)	1990	(87.63)	
Energy intake							
1Q	1110	(25.80)	147	(13.24)	963	(86.76)	0.5965
2Q	1085	(25.21)	136	(12.53)	949	(87.47)	
3Q	1057	(24.56)	151	(14.29)	906	(85.71)	
4Q	1051	(24.42)	149	(14.18)	902	(85.82)	
Current smoking status							
No	3548	(82.45)	462	(13.02)	3086	(86.98)	0.0285
Yes	755	(17.55)	121	(16.03)	634	(83.97)	
Alcohol use							
No	2070	(48.11)	276	(13.33)	1794	(86.67)	0.6911
Yes	2233	(51.89)	307	(13.75)	1926	(86.25)	
Depressive symptoms							
No	4015	(93.31)	546	(13.60)	3469	(86.40)	0.7188
Yes	288	(6.69)	37	(12.85)	251	(87.15)	
Number of chronic diseases ^a^							
0	3409	(79.22)	403	(11.82)	3006	(88.18)	<0.0001
1	735	(17.08)	140	(19.05)	595	(80.95)	
≥ 2	159	(3.70)	40	(25.16)	119	(74.84)	
Total	4303	(100.00)	583	(13.55)	3720	(86.45)	

^a^Number of chronic diseases includes cancer, stroke, myocardial infarction, angina, arthritis, rheumatoid arthritis, asthma, thyroid gland disorder, chronic renal failure, and hepatitis B

Table 2 presents the estimated odds ratios (ORs) from the multiple logistic regression analysis. Men had a nearly twofold higher risk of MS compared to women (OR: 1.92, 95 % confidence interval [CI]: 1.52–2.42). Regarding sitting time, the risk of MS was greater among those who sat for >7 h/day relative to those who sat for ≤7 h/day (OR: 1.21, 95 % CI: 1.00–1.46). Regarding occupation, office workers had a twofold higher risk of MS than did AFF workers (OR: 2.01, 95 % CI: 1.26–3.22). Regarding educational levels, lower levels of education resulted in a greater risk of MS than college level or higher (elementary school, OR: 2.54, 95 % CI: 1.79–3.59; middle school, OR: 1.61, 95 % CI: 1.11–2.33; high school, OR: 1.43, 95 % CI: 1.07–1.89).

**Table 2:** Results of multiple logistic regression analysis

	Metabolic syndrome	
	OR	95 % CI
Sitting time (hours)		
≤ 7	1.00	
> 7	1.21	(1.00–1.46)
Sex		
Male	1.92	(1.52–2.42)
Female	1.00	
Age (year)		
19–29	0.26	(0.13–0.50)
30–39	1.00	
40–49	1.32	(0.92–1.90)
50–59	1.64	(1.14–2.36)
60–69	2.19	(1.50–3.18)
70+	1.78	(1.17–2.70)
Household income level		
Low	1.16	(0.85–1.60)
Lower middle	1.07	(0.81–1.42)
Upper middle	1.15	(0.88–1.50)
High	1.00	
Educational levels		
Elementary school	2.54	(1.79–3.59)
Middle school	1.61	(1.11–2.33)
High school	1.43	(1.07–1.89)
Above college	1.00	
Occupation		
Office work	2.01	(1.26–3.22)
Sales and Service	1.61	(1.00–2.60)
Agriculture, forestry and fishery	1.00	
Machine fitting and simple labor	1.61	(1.06–2.44)
Unemployed, housewife or students	1.71	(1.14–2.55)
Physical activity		
Under meet recommended guideline	1.03	(0.85–1.24)
Meet recommended guideline	1.00	
Energy intake		
1Q	1.00	
2Q	1.00	(0.77–1.31)
3Q	1.18	(0.90–1.53)
4Q	1.23	(0.93–1.63)
Depressive symptoms		
No	1.00	
Yes	1.22	(0.83–1.79)
Current Smoking status		
No	1.00	
Yes	1.07	(0.83–1.38)
Alcohol use		
No	1.00	
Yes	1.11	(0.90–1.35)
Number of chronic diseases ^a^		
0	1.00	
1	1.35	(1.07–1.70)
≥ 2	1.82	(1.22–2.71)

^a^Number of chronic diseases, including cancer, stroke, myocardial infarction, angina, arthritis, rheumatoid arthritis, asthma, thyroid gland disorder, chronic renal failure, and hepatitis B

*OR* odds ratio, *CI* confidence interval

**Fig. 1:**
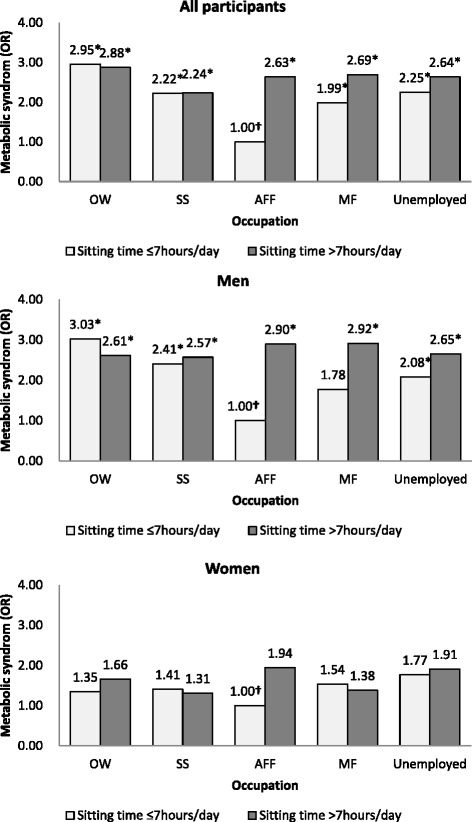
Results of a combined analysis of sitting time, occupation, and metabolic syndrome. * *p* <0.05. *OR* odds ratio. † Reference category. ‡ *OW* office work, *SS* sales and service, *AFF* agriculture, forestry, and fishery, *MF* machine fitting and simple labor. ¶ Adjusted for age, household income, educational level, physical activity, energy intake, depressive symptoms, smoking, alcohol use, and chronic diseases

A combined analysis was conducted after observing an effect of the interaction between sitting time and occupation on MS. Figure 1 depicts a subgroup analysis in which multiple logistic regression was performed to assess the relationship between the combined variables (sitting time × occupation) and MS according to physical activity. Notably, all participants who sat for >7 h/day, regardless of occupation, had a higher risk of MS relative to those who sat for ≤7 h/day (>7 h/day × OW, OR: 2.88, 95 % CI: 1.61–5.13; >7 h/day × SS, OR: 2.24, 95 % CI: 1.11–4.50; >7 h/day × AFF, OR: 2.63, 95 % CI: 1.24–5.61; >7 h/day × MFF, OR: 2.69, 95 % CI: 1.54–4.71; >7 h/day × unemployed, OR: 2.64, 95 % CI: 1.57–4.42; Fig. 1 and Appendix). In particular, men who sat for >7 h/day and reported office work or machine fitting as their occupation were significantly more likely to report MS relative to those who sat for ≤7 h/day and worked in an AFF position (>7 h/day × OW, OR: 2.41, 95 % CI: 1.05–5.51; >7 h/day × MF, OR: 2.92, 95 % CI: 1.43–5.93); no statistically significant association was observed for women.

## Discussion

To the authors’ knowledge, this study was among the first to consider the effect of overall sitting time and occupation on the risk of MS in a Korean population. This study, which was based on a survey with a cross-sectional design, identified a positive association between a long sitting time (>7 h/day) and an increased risk of MS, independent of physical activity or other potential confounders. In addition, office workers had a twofold higher risk of MS relative to those who worked in AFF occupations; similarly, sales and service workers and machine fitting and simple laborers had a 1.6-fold higher risk of MS relative to those with AFF occupations. Moreover, lower educational levels were related to a higher risk of MS; particularly, people whose final educational level was elementary school had a 2.5-fold higher risk of MS than those who had graduated from college or a higher institution.

The results of the present study were consistent with those of previous studies, which also found a negative relationship between longer periods of sitting and MS. Specifically, MS and related metabolic risk factors have been directly associated with sitting time and/or positively associated with a low level of physical activity [29]. Studies on prolonged television and computer use concluded that excessive sitting could more than double the risk of MS; for example, Dunstan and colleagues observed a 26 % increase in the prevalence of MS in women for each 1-h increase in television use per day. As in our study, a study of a middle-aged French population identified a positive association between sedentary behaviors such as television or computer use and MS. Furthermore, a study by Park and colleagues described a significant association between the type of occupation and risk of MS in a Korean population [30]; specifically, disparities in MS prevalence were observed among occupations, suggesting that white collar and unemployed workers were at a greater risk of developing MS than blue collar workers, similar to the findings of the present study. Additionally, Myong and colleagues classified occupations as manual (e.g., office workers, technicians, and sales and service workers) or non-manual (e.g., agriculture, forestry, fishery, mining, and manufacturing workers) and found that younger workers with manual occupations and older workers with non-manual occupations had the highest risk of MS [31]. Although those results were similar to the results for male subjects in the present study, the latter used a different occupational classification and did not stratify the analysis according to age. In another study, Bradshaw and colleagues described an association between higher education level and low prevalence of MS in a US community [32]. This finding was similar to the present study and implies that those with lower education levels may be vulnerable to MS due to the greater likelihood of performing simple or repeated manual labor or receiving a lower salary, which would cause them to tend to work longer hours. As a result, they may be prone to having less leisure time or poor nutritional habits; therefore, they have a higher risk of MS compared to highly educated people.

Given the statistically significant interaction between occupation and sitting time that was identified in the present study, we also conducted a combined analysis. When compared with participants who sat for ≤7 h/day and had an AFF occupation, all other participants had a higher risk of MS. In addition, after excluding office workers, all participants and particularly male participants who sat for >7 h/day remained at a higher risk of MS relative to those who sat for ≤7 h/day, regardless of occupation. Interestingly, office workers who sat for ≤7 h/day had a higher risk of MS relative to those who sat for >7 h/day, in contrast to the findings of previous studies. This discrepancy may be attributable to multiple factors. First, our sample size was very small after stratification according to several variables; therefore, we might have overestimated the frequency of participants with MS among those with a reported sitting time of ≤7 h/day. Second, among men, older workers with higher professional positions tended to sit for shorter periods of time relative to younger and lower-grade workers due to the tendency of older professionals to perform active work, such as meetings or external activities (e.g., drawing contracts), rather than paperwork at a desk. However, these older workers with higher positions might also be more likely to exhibit negative health behaviors such as smoking or binge drinking, leading to the above association of a lower sitting time with a higher risk of MS. Further research should be conducted to address this potential issue.

Several studies have described the potential mechanisms underlying the inverse relationships of sitting time and occupation with MS. First, the positive relationship between sitting time and MS risk could be attributed to the replacement of physical activity by sitting time, thus reducing energy expenditure [33]. Although the present study did not evaluate the effect of sitting time according to physical activity, previous studies have indicated that physical activity is associated with MS [34]. Moreover, the present study determined that there was no statistically significant association between physical activity or energy intake and MS, which implies that sedentary behavior might be a significant factor for the increase of MS. Therefore, moving or standing within the workplace is important, as it could increase energy expenditure and may reduce the risk of MS. Second, a prolonged sitting time might provide more opportunities to consume food, thus increasing the total energy intake during sedentary activities (e.g., television use); accordingly, the prevalence of overweight and obesity, which are known to be significant determinants of MS, may increase [35, 36]. Third, several mechanisms that might be complex in nature and possibly reciprocal in terms of influence [37] have been proposed. In a study by Hamilton and colleagues [29, 38, 39], a loss of local contractile stimulation, which generally occurs while sitting or lying down, was found to repress the activity of skeletal muscle lipoprotein lipase, which is involved in the uptake of triglycerides and free fatty acids into the skeletal muscle, as well as high-density lipoprotein cholesterol production. Local muscle shrinkage might also reduce glucose uptake via the blunted translocation of GLUT-4 glucose transporters to the skeletal muscle cell surface [29, 40]. Increases in the levels of glucose, triglycerides, and free fatty acids could result in an excess of free radicals and thus provoke a biochemical cascade of inflammation, endothelial dysfunction, hypercoagulability, and increased sympathetic activity. Over time, such events could create a milieu that favors the development of cardiovascular risk factors [41]. Notably, the repression of lipoprotein lipase activity is not easily observed in experimental animal models of low-intensity activities such as standing or walking.

Recent studies have identified that many working adults spend considerable amounts of sedentary time in the workplace; for example, Australian adults spend an average of 57 % of their working hours in sedentary behaviors, and US adults spend 7.3–7.9 h/day in sedentary behaviors [15, 16], indicating that during workdays, at least a portion of this sedentary time occurs during working hours [17, 18]. Excessive sitting and insufficient physical activity during working hours has been increasingly recognized as a public health risk [42, 43], leading many researchers to propose recommendations for sufficient workplace physical activity such as using sit-stand desks [44, 45] and participating in workplace healthpromotions during lunch or other breaks from work [19–21]. Unfortunately, in South Korea, men with a higher socioeconomic status (e.g., white collar workers) tend to exhibit more sedentary behaviors and have more opportunities to consume richer foods and alcohol relative to physical workers [46]. Moreover, the lifestyles of Korean workers, whose labor hours are among longest in OECD countries, place them at an increased risk of MS. These phenomena may result in not only a higher burden of disease but also corporate economic losses resulting from increased absences due to sickness. Therefore, further studies should focus on the relationship of occupation and sedentary behavior with MS in the context of physical activity.

We note that this study had several limitations. First, this was a cross-sectional study; therefore, we could not explain whether sitting for long periods was the cause or consequence of MS. Reverse causality is therefore recognized as a potential confounder for the observed association. Second, work and leisure time were not separated by the KNHANES; as a result, both were included as “sitting time” in this study, and we could not ascertain whether work-related or leisure-related sitting time affected MS. Therefore, further research should be conducted to overcome this limitation and yield a more precise and elaborate analysis. Third, despite the decreased effect of confounding factors, residual confounders, such as genetic factors, might nevertheless influence MS. Fourth, although this study included a nationally representative sample of South Korean adults, the small MS event sample size, which was obtained during a period of only 1 year, was limited with respect to more stratified analyses. Nevertheless, this study was unique as it was the first to examine the relationships between sitting time, occupation, and MS in a representative national sample of the South Korean population. Moreover, this study used objective measurements of MS, as well as risk factors of MS identified via health examinations, such as repeated blood pressure analyses with a mercury sphygmomanometer and blood testing of fasting plasma glucose and lipid concentrations after a 12-h overnight fast.

## Conclusions

This study identified a correlation between excessive sitting-time and an increased risk of MS. Moreover, long periods of sitting combined with a sedentary occupation were associated with a high risk of MS. These findings accentuate the importance of reducing the overall sitting time and increasing physical activity in the workplace and highlight the need for public policies to address these issues and thus alleviate the burden of MS in terms of fiscal health premiums, corporate economic losses, and negative health outcomes related to workers’ sedentary behaviors.

## Appendix

**Table 3:** Results of combined analysis of sitting time, occupation, and metabolic syndrome

	Metabolic syndrome (total) (All participants)		Metabolic syndrome (men) (men)		Metabolic syndrome (women) (women)	
	OR	95 % CI	OR	95 % CI	OR	95 % CI
Office work × Sitting (≤7 h)	2.95	(1.62–5.38)	3.03	(1.43–6.42)	1.35	(0.43–4.22)
Sales and Service × Sitting (≤7 h)	2.22	(1.23–4.01)	2.41	(1.05–5.51)	1.41	(0.59–3.33)
Agriculture, forestry, and fishery × Sitting (≤7 h)	1.00		1.00		1.00	
Machine fitting and simple labor × Sitting (≤7 h)	1.99	(1.17–3.38)	1.78	(0.89–3.57)	1.54	(0.67–3.51)
Unemployed, housewife, or student × Sitting (≤7 h)	2.25	(1.35–3.74)	2.08	(1.03–4.21)	1.77	(0.83–3.76)
Office work × Sitting (>7 h)	2.88	(1.61–5.13)	2.61	(1.25–5.47)	1.66	(0.59–4.68)
Sales and Service × Sitting (>7 h)	2.24	(1.11–4.50)	2.57	(1.02–6.46)	1.31	(0.44–3.88)
Agriculture, forestry, and fishery × Sitting (>7 h)	2.63	(1.24–5.61)	2.90	(1.04–8.04)	1.94	(0.63–5.95)
Machine fitting and simple labor × Sitting (>7 h)	2.69	(1.54–4.71)	2.92	(1.43–5.93)	1.38	(0.54–3.53)
Unemployed, housewife, or student × Sitting (>7 h)	2.64	(1.57–4.42)	2.65	(1.30–5.41)	1.91	(0.89–4.11)

Adjusted for age, household income, educational level, physical activity, energy intake, depressive symptoms, smoking, alcohol use, and chronic diseases

*OR* odds ratio, *CI* confidence interval

## Abbreviations

AFF: agriculture, forestry and fishery
CI: confidence interval
KNHANES: Korean National Health and Nutrition Examination Survey
MF: machine fitting and simple labor
MS: metabolic syndrome
NHANES: the National Health and Nutrition Examination Survey
NHIS: the National Health Interview Survey
OECD: Organization for Economic Co-operation and Development
OR: odds ratio
OW: office work
PA: physical activity
SS: sales and services
